# Enhanced radiosensitizing by sodium glycididazole in a recurrent esophageal carcinoma tumor model

**DOI:** 10.18632/oncotarget.19151

**Published:** 2017-07-10

**Authors:** Peipei Wu, Jing Liu, Xiaorong Sun, Xiaolin Li, Ligang Xing, Jinming Yu

**Affiliations:** ^1^ Department of Radiation Oncology, Shandong Key Laboratory of Radiation Oncology, Shandong Cancer Hospital Affiliated to Shandong University, Shandong Academic of Medicine Science, Jinan 250117, Shandong, China; ^2^ Department of Oncology, Jining No.1 People's Hospital, Jining 272011, Shandong, China; ^3^ Department of Radiology, Shandong Key Laboratory of Radiation Oncology, Shandong Cancer Hospital Affiliated to Shandong University, Shandong Academic of Medicine Science, Jinan 250117, Shandong, China

**Keywords:** esophageal cancer, sodium glycididazole, radiosensitizer, tumor bed effect, hypoxia

## Abstract

Re-irradiation is challenging for esophageal cancer patients with local-regional recurrence after initial radiotherapy. The purpose of this study is to establish a recurrent esophageal tumor model and investigate radiosensitizing effects of sodium glycididazole (CMNa). Tumor models were established by pre-irradiation (0 Gy, 10 Gy or 20 Gy) to the right hind leg of the nude mice 24 hours before tumor transplantation (ECA109 human esophageal carcinoma cells). Tumor growth curves were analyzed. Hypoxic microenvironment was exhibited in tumor frozen slides stained for pimonidazole, Hoechst 33342, hematoxylin-eosin and CD34. Mice bearing primary (0 Gy pre-irradiation) and recurrent (10 Gy pre-irradiation) tumors were randomized into control (no treatment), radiation (30 Gy in 3 weekly fractionations), or radiation combined with CMNa (1 mmol/kg *i.p*. injected 60 min before radiation) respectively. The data showed tumors from 10 Gy and 20 Gy pre-irradiated sites grew significantly slower than those in the 0 Gy pre-irradiated group. The recurrent xenograft tumors showed increased necrotic fractions, decreased micro-vascular density, increased pimonidazole-positive fraction, and decreased Hoechst-positive fraction. In the primary xenograft tumors, CMNa adding to radiation did not lead to significant tumor growth delay than radiation alone. However, for the recurrent tumor model, the growth rate was remarkably reduced as CMNa combined with radiation as comparison with radiation alone. In conclusion, the recurrent esophageal xenograft model with tumor bed effect was successfully established characterized by slow growth, increased hypoxia fraction and decreased blood flow. Significant radiosensitization by CMNa was demonstrated in the recurrent model.

## INTRODUCTION

Radiotherapy is the mainstay in management of esophageal carcinoma [1, 2]. However, local-regional recurrence after radiotherapy was about 40∼50% despite of advancing radiation techniques and advanced chemotherapy/targeted therapy [3, 4]. Salvage re-irradiation with/without chemotherapy is an important choice, but it is challenging because normal tissue already got high-dose irradiation during first setting of treatment. More importantly, the radiobiological characteristics of recurrent tumor after radiotherapy are different from the primary tumors [5, 6].

To elucidate its biology, serial xenograft models for recurrent tumors were established. Tumor transplanted into pre-irradiated tissue has been frequently used as recurrent xenograft tumor models [5–8]. Recurrent tumors were often found to be resistant to re-irradiation, resulted in poor disease control and limited survival [6, 9, 10]. This could be due to the condition known as “tumor bed effect” (TBE), which showed tumors in pre-irradiated beds commonly characterized by reduced blood perfusion, extensive necrosis, and elevated hypoxic fractions. Existed study [9] showed that TBE appeared at single 5-30 Gy irradiation or 40-60 Gy total dose in a conventional fractionation mode dependent on different tumor types. However, recurrent tumor model in esophageal cancer was not well-documented.

As the reported higher hypoxia fraction in the recurrent tumors, it is possible to improve the radiation effect using hypoxia radiosensitizer. However, few related studies were reported. Sodium glycididazole (C18H22N7NaO10·3H2O), abbreviated CMNa, is a new nitroimidazole compound independently developed in China. Experimental and clinical studies [11–20] reported significant radiosensitizing effects of CMNa in solid tumors without adverse influences on normal tissues. The aims of present preclinical study are to: (1) establish a recurrent esophageal tumor model, (2) define the hypoxic microenvironment of the established recurrent tumor model, and (3) investigate the radiosensitizing effects of CMNa in the recurrent tumor model.

## RESULTS

### Growth pattern in the recurrent esophageal tumor model

**Figure 1:**
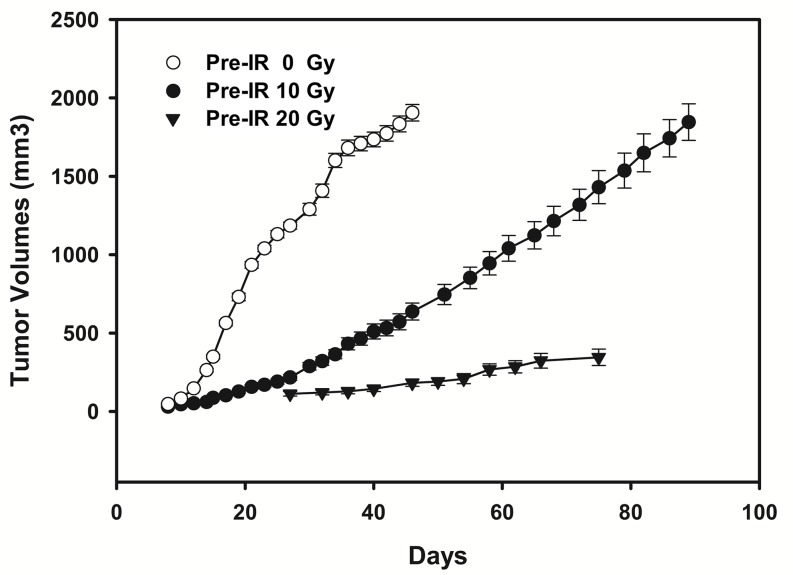
Tumor growth curves of mice (*n* = 10/group) inoculated with ECA109 cells 24 hours after the 0 Gy, 10 Gy and 20 Gy pre-irradiation to the right hind leg Tumors from 10 Gy and 20 Gy pre-irradiated mice grew significantly slower than those in 0 Gy pre-irradiated group (*P*=0.005 for 10 Gy *vs* 0 Gy pre-irradiation; *P*<0.001 for 20 Gy *vs* 0 Gy pre-irradiation). As for exposure dose, growth delay was much more significant in mice with 20 Gy pre-radiation than those with 10 Gy (*P*=0.002).

The primary (0 Gy pre-irradiation) and recurrent (10 Gy or 20 Gy pre-irradiation) esophageal tumor models were established successfully with tumor formation rate of 95%. The TBE was clearly seen through comparison of tumors growth pattern between 0 Gy pre-irradiation and 10 or 20 Gy pre-irradiation groups (Figure 1). Tumors from pre-irradiated mice grew significantly slower than those in the un-irradiated group (*P*=0.005 for 10 Gy *vs* 0 Gy pre-irradiation, *P*<0.001 for 20 Gy *vs* 0 Gy pre-irradiation). As for exposure dose, growth delay was much more significant in mice with 20 Gy pre-radiation than those with 10 Gy (*P*=0.002). The time intervals needed for tumor grown to a 10 mm average diameter were 17 days and 33 days for 0 Gy and 10 Gy pre-irradiated groups, respectively. Tumors in mice receiving 20 Gy pre-irradiation failed in growing large enough to calculate the above time interval. Therefore, the 10 Gy pre-irradiation group was selected for further radiosensetizing experiments.

### Hypoxic characteristics in the recurrent esophageal tumor model

**Figure 2:**
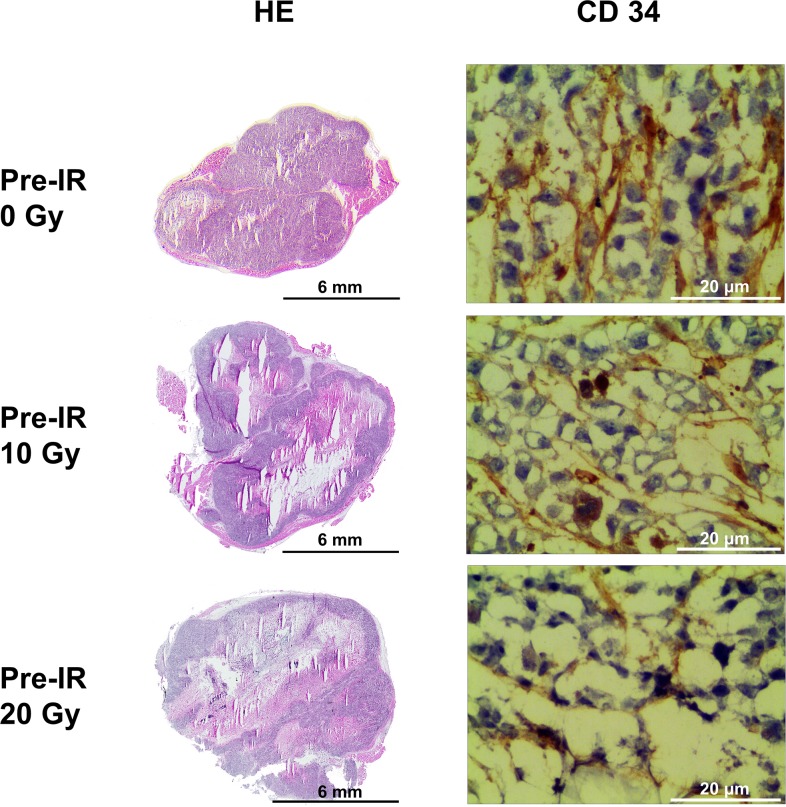
HE staining and CD34 immunohistochemical staining of tumors in the 0 Gy, 10 Gy and 20 Gy pre-irradiation models The necrotic fractions derived from HE staining increased as pre-irradiation dose increasing (*P*<0.001). Specific staining of capillary-like vessels by anti-CD34 showed significant decrease of intra-tumoral microvessel density (MVD) in 10 Gy and 20 Gy pre-irradiation compared with 0 Gy pre-irradiation (*P*=0.016). MVD in tumor was negatively correlated with the necrotic fractions (r=-0.751,*P*=0.005).

**Figure 3:**
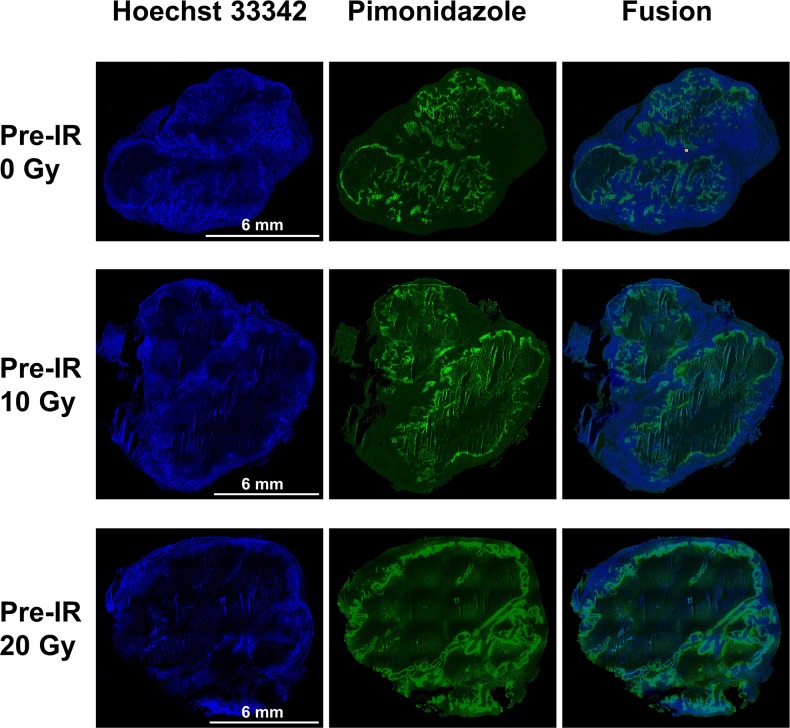
Pimonidazole, Hoechst 33342 and fusion images of tumors in the 0 Gy, 10 Gy and 20 Gy pre-irradiation models Pimonidazole binding was primarily confined to peri-necrotic regions while Hoechst 33342 binding located at outer edge of tumor. There was a tendency for intratumoral regions staining positive for pimonidazole or for Hoechst 33342 to be mutually exclusive. A significant higher pimonidazole-positive fraction and lower Hoechst-positive fraction were seen in tumors of 10 Gy and 20 Gy pre-irradiation models than 0 Gy pre-irradiation models (*P*=0.010, *P*=0.018).

The characteristics of hypoxia (pimonidazole), tumor blood perfusion (Hoechst 33342), microvessel formation (CD34) and morphology [hematoxylin-eosin (HE)] of tumors were examined and compared (Figures 2 and 3) in 0 Gy, 10 Gy and 20 Gy pre-irradiated mice. Necrosis was clearly seen in tumors central area with HE staining. The necrotic fractions were 11.78% ± 2.99% in 0 Gy pre-irradiation group, while increased significantly to 32.78% ± 7.27% in 10 Gy pre-irradiated tumors, and 55.52% ± 14.28% in 20 Gy pre-irradiated tumors, *P*<0.001. Specific staining of capillary-like vessels by anti-CD34 was shown in tumor tissues. Pre-irradiation resulted in significant decrease of intra-tumoral microvessel density (MVD) compared with the control. The MVD were 36.67 ± 5.61 vessels/field, 25.50 ± 4.55 vessels/field, and 11.96 ± 2.83 vessels/field in 0 Gy, 10 Gy and 20 Gy pre-irradiation models, respectively (*P*=0.016). Analysis of all tumors showed the MVD in tumor was negatively correlated with the necrotic fractions (r=-0.751, *P*=0.005).

Pimonidazole binding was primarily confined to peri-necrotic regions while Hoechst 33342 binding located at outer edge of tumor. There was a tendency for intratumoral regions staining positive for pimonidazole or for Hoechst 33342 to be mutually exclusive. The pimonidazole-positive fraction (PPF) and Hoechst-positive fraction (HPF) were calculated for each group. A significant higher PPF and lower HPF were seen in tumors of 20 Gy and 10 Gy pre-irradiation models than 0 Gy pre-irradiation models (PPF: 46.55% ± 15.80%, 22.68% ± 7.80% and 4.92% ± 1.69%, *P*=0.010; HPF: 38.98% ± 11.79%, 59.65% ± 17.74% and 78.96% ± 23.53%, *P*=0.018. There were statistical differences between 20 Gy and 10 Gy pre-irradiation groups as well, which illustrated that the extent of hypoxia and blood perfusion was also dose-dependent.

### Radiosensitizing effect of CMNa in the primary and recurrent tumor models

**Figure 4:**
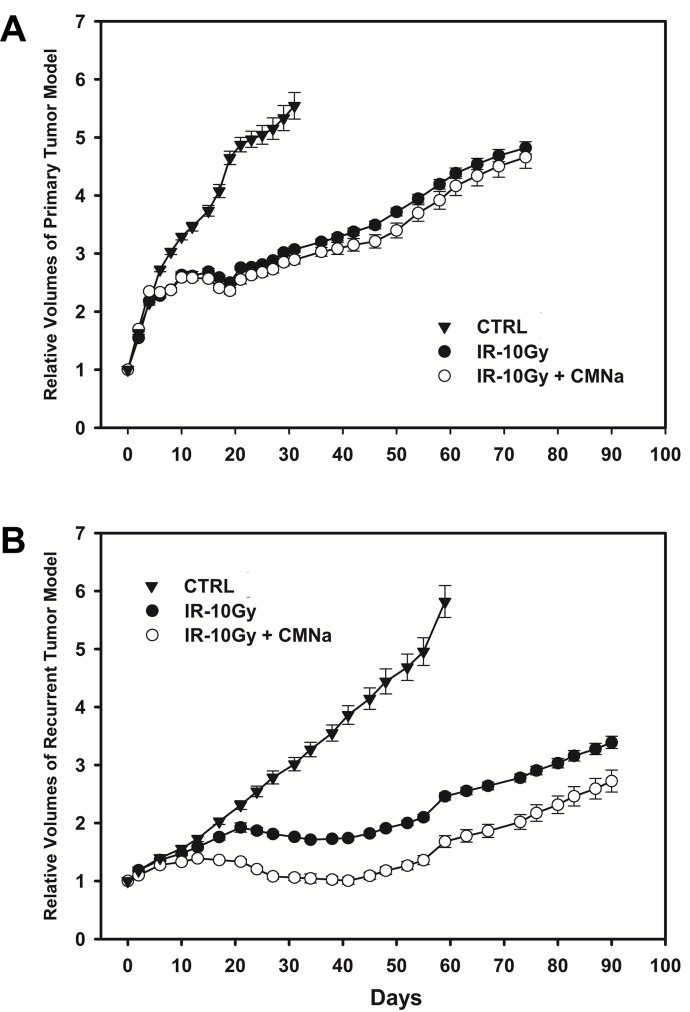
Tumor growth curves of mice with no treatment, irradiation-alone and CMNa+irradiation in the primary and the recurrent esophageal tumor models **(A)** Tumor growth curves of mice with no treatment, radiation alone and CMNa + radiation in the primary esophageal tumor model. Radiation alone inhibited tumor growth (*P*<0.001). There was no significant difference in growth between the radiation alone and CMNa + radiation (*P*=0.285). **(B)** Tumor growth curves of mice with no treatment, radiation-alone and CMNa + radiation in the recurrent esophageal tumor model. Radiation alone also inhibited tumor growth (*P*<0.001). The growth rate was remarkably reduced under CMNa + radiation as comparison with the radiation alone (*P*=0.032) for tumors growing in the pre-irradiation group.

As shown in Figure 4, irradiation alone inhibited tumor growth in both the primary (*P*<0.001) and recurrent tumor models (*P*<0.001). There was no significant difference in growth between radiation alone and CMNa + radiation (*P*=0.285) in the primary esophageal xenograft tumors. In contrast, for tumors growing in the recurrent tumor models, the growth rate was remarkably reduced under CMNa + radiation as comparison with the radiation alone (*P*=0.032).

The tumor inhibition rate of CMNa + radiation in the recurrent tumor model was 40.81% as peak value at 30 days after last fractionation of radiation. However, the maximal inhibition rate of by CMNa + radiation was only 8.05% at 35 days after treatments in the primary tumor model. The relative growth delay due to the CMNa + radiation treatment was much longer than radiation alone in the recurrent tumor model (*P*<0.001). The relative growth delay times were 7.46 ± 0.58, 28.34 ± 4.41, and 35.48 ± 4.56 days for blank treatment, radiation alone and CMNa + radiation groups in the primary tumor model. However, the relative growth delay times were 28.52 ± 3.41 and 85.72 ± 8.06 days for blank treatment and radiation alone in the recurrent tumor models. For the CMNa + radiation tumors in the recurrent tumor models, tumors grown to 2.72 times of initial volume at 97 days after last treatment when observation was ended.

## DISCUSSION

High hypoxic fraction was often seen in recurrent tumors with subsequent poor radiosensitivity. The hypoxic microenvironment and radiosensitizing role of CMNa in pre-clinical models of recurrent primary tumors were studied in the present work by using ECA109 human esophageal xenografts growing in un-irradiated and pre-irradiated beds in BALB/c-nu/nu mice. Our *in vivo* experimental exploration succeeded in establishing recurrent esophageal xenograft tumor model, which characterized with significant hypoxic microenvironment. More importantly, promising radioenhancing roles of CMNa in established recurrent tumor model was demonstrated and provided theoretical basis for application of CMNa for recurrent tumors in further clinical trials.

Tumor cells transplanted in pre-irradiated beds of nude mice are frequently used as experimental models of recurrent primary tumors in humans [5–8]. The TBE is considered to be mainly caused by radiation induced injury to the host vasculature and resulted impaired neovascularization. The pre-irradiation dose needed to cause TBE varies according to different tumor types. In our study, 10 Gy and 20 Gy pre-irradiation to tumor beds resulted increased hypoxic fraction, increased necrotic fraction and decreased microvascular density. The hypoxic microenvironment is associated with decreased radiosensitivity and thus remarkable radiosensitizing effect by hypoxic sensitizer. Chen et al [5] reported similar TBE and resistance to re-irradiation and antiangiogenic therapy of sunitinib by implanting mouse prostate C1 tumors to un-irradiated or pre-irradiated tissues and examining vascularity and hypoxia by immunohistochemistry. Additionally, recent researches [7, 21, 22] had shown that tumor hypoxia may promote metastasis through multiple signal pathways. Rofstad et al [7] showed that pre-irradiation generated environmental hypoxia is associated with enhanced tumor invasion and metastasis which leaded by transcriptional activation of metastasis-promoting genes including the receptor for the urokinase-type plasminogen activator receptor. In Rezaeian's study [21], the hypoxia-responsive TRAF6 overexpression promotes breast cancer progression and metastasis to lung and spinal bone, and targeting of TRAF6 reduces breast cancer metastasis, opening up opportunities for therapeutic intervention. Mao et al [22] shows that in HCC cells, hypoxia elevates expression of Cav1, which then acts through the calcium-binding protein S100P to promote metastasis. Further researches about the microenvironment of these recurrent tumor models will be helpful to developing targeted therapy in addition to hypoxia radiosensitizers.

The nitroimidazoles, the most commonly studied hypoxic radiosensitizer, that sensitize radioresistant hypoxic cells to ionizing radiation are believed to mimic oxygen. Oxygen radically alters the pattern of base damage and significantly enhances the level of strand cleavage, especially strand breaks with phosphoglycolate termini. The presence of nitroimidazoles under anoxic conditions does not increase the level of strand breakage than in aerobic conditions, but, like oxygen, significantly enhances the formation of 3′-phosphoglycolate end groups [23]. At a clinically acceptable toxicity level, an expected oxygen enhancement ratio of, at the most, 1.5 to 2.0 can be theoretically achieved by nitroimidazoles [24]. CMNa belongs to 5-nitroimizazole derivatives, which increases the sensitivity of hypoxic tumor cells to radiotherapy by settling down the molecular damage to DNA and preventing potentially lethal damage repair and sublethal damage repair of DNA molecule with less severe toxicity [25]. CMNa has been clinically applied for radiosensitization with improving treatment response without additional adverse events in head and neck cancer and esophageal cancer. Zeng et al [11] compared radiotherapy with and without CMNa in locally advanced laryngeal squamous cell carcinoma. Overall response rate was significantly improved from 58.33% to 88.89%. Similarly, He et al [12] reported that radiosensitivity of CMNa in locally advanced nasopharyngeal carcinoma receiving concurrent radiotherapy and cisplatin-based chemotherapy. Higher complete response rate of 93.33% were obtained in the CMNa group compared with 73.33% in the radio-chemotherapy controlled group. Additionally, in NSCLC patients with multiple brain metastases, CMNa also played effective radiation-enhancing roles, resulted in improved CNS disease control rate (90.6% *vs* 65.6%, *P*=0.016), extended median CNS progression-free survival time (7.0 months *vs* 4.0 months, *P*=0.038), and well toleration [19].

CMNa also been reported to be effect in recurrent tumors. Cheng et al [26] reported 46 patients with recurrent esophageal cancer after complete response to radical radiotherapy. The short-term response rates were 74% versus 44% in the CMNa combination group and re-radiation alone group with statistical significance. The 1-, 2- and 3-year local control rates were 65%, 39%, 30% in the CMNa combination group and 44%, 17%, 9% in the re-irradiation alone group (*P*<0.05). Liang et al [27] reported similar radio-enhancing effects of CMNa in locally recurrent nasopharyngeal carcinoma patients with significantly improved local tumor regression rate and equally toxicities. Well-designed large scale multicenter clinical trials on improving therapeutic efficacy of recurrent tumors were still warranted.

Limitations existed in the present study. Firstly, hypo-fractionated radiotherapy with 30 Gy in 3 fractionations at 10 Gy/fraction were applied and offered good response in radiation-alone group. Secondly, only one esophageal carcinoma cell line of ECA109 was applied. Additionally, single CMNa concentration 1 mmol/kg was used which has been verified previously [28]. Future studies would be necessary to be launched to improve the recurrent esophageal tumor model, taking into account the radiotherapy protocols that are used in patients with esophageal cancer. Conventional fractionation schedule instead of hypo-fractionation should be applied during treatment in xenograft tumor nude mice models. Additional esophageal squamous carcinoma cell lines including TE-1 and KYSE30 may be cultured to repeat the experiment protocol to validate the preliminary results. Moreover, pharmacokinetic characteristics and tissue distribution of CMNa after intravenous administration were explored in mice xenografts models of ECA109, FaDu and A549 by investigators from our study group (the article has been accepted but not published online). As a reference, different CMNa concentrations may be administrated in future protocols.

In conclusion, the present preclinical study demonstrated that the human esophageal xenograft recurrence tumor model with tumor bed effect could be established by a single dose of 10 Gy or 20 Gy pre-irradiation before tumor transplantation. After pre-irradiation, the tumors grew much slower with increased hypoxia fraction, decreased blood flow, and decreased radiation sensitivity. Radiosensitization with CMNa delayed tumor growth significantly. It is suggested that hypoxia radiosensitizer as CMNa might improve tumor control for patients with in-field recurrences after primary radiotherapy.

## MATERIALS AND METHODS

### Cell culture

The human esophageal squamous carcinoma cell line (ECA109) was purchased from Chinese Academy of Sciences Shanghai Institute of Cell Bank. The cells were cultured in RPMI 1640 medium (Gibco, USA) supplemented with 10% fetal bovine serum (Gibco, USA), penicillin (100 units/ml), and streptomycin (100 μg/ml).

### Establishment of recurrent tumor model

The animal experimental protocols has been approved by the institutional research ethics committee of Shandong Cancer Hospital and Institute within which the work was undertaken and that it conforms to the provisions of the Declaration of Helsinki in 1995 (as revised in Edinburgh 2000). All experiments were performed using 6- to 8-week-old female athymic BALB/c nude mice purchased from Beijing Hua Fukang pathogen-free animal breeding facility (approval no. SCXK [Jing] 2009-0008). Nude mice were kept in accordance with institutional guidelines in specific pathogen free units. Tumors were initiated by injecting 5×10^6^ cells per mouse in 100 μL PBS medium s.c. in the right hind leg of mice.

Nude mice were randomly divided into 0 Gy (un-irradiation group), 10 Gy and 20 Gy pre-irradiation groups. Mice were fixed with the right hind leg irradiated (0, 10 or 20 Gy) using an X-ray unit (X-Rad 225, PXI, USA) at 225 kV and 13.30 mA 24 hours before tumor transplantation. Mice were inspected daily and tumors measured with a caliper twice per week. Tumor volume was calculated as π/6×a×b^2^, where “a” is the longest and “b” is the shorter of two orthogonal diameters.

### Tumor sample preparation and Immunohistochemistry staining

Six mice in each group were sacrificed when tumor grew to approximately 10 mm in diameter. The hypoxic cell marker pimonidazole hydrochloride (16 mg/mL in saline; 80 mg/kg; Chemicon International) was *i.p*. injected 2 h before sacrifice. The Hoechst 33342 (5 mg/mL in saline; 25 mg/kg; Sigma-Aldrich) was injected via tail vein 1 min before sacrifice. Immediately after animal sacrifice, tumors were removed quickly and embedded into optimal cutting medium (OCT 4583, Sakura Finetek), and frozen on dry ice. Sets of 10 contiguous 8μm thick tissue sections were prepared for further analysis.

Frozen slides were air dried, fixed in ice-cold acetone for 20 min, and incubated with SuperBlock (Pierce Biotechnology) at room temperature for 30 min. Sections were then incubated with FITC-conjugated anti-pimonidazole monoclonal antibody (Chemicon International) diluted 1:50 in blocking solution for 1 h at room temperature. Images were acquired at using Nikon H600L ECLIPSE 90i fluorescence microscope (Nikon, Japan) equipped with a motorized stage, NIS-Elements and Image J software. Hoechst 33342 and pimonidazole were imaged using blue and green filters respectively.

Adjacent continuous tumor sections were used for standard HE staining and CD34 immunohistochemistry and imaged by light microscopy. Frozen sections were stained with primary anti-CD34 antibodies (Abcam, Cambridge, USA), washed, and then a brown precipitate was developed at sites of primary antibody binding through use of a peroxidase-conjugated second step antibody and a 3,3-diaminobenzidine reagent.

### Immunohistochemistry image analysis

The images obtained were visually scored blinded by two independent researchers (Jing Liu and Peipei Wu) with good inter-observer reproducibility. Images with pimonidazole and Hoechst staining were manually matched to the HE staining images based on the position of tissue edges and necrotic areas by Adobe Photoshop (Adobe, San Jose, CA). Tumor area was defined by contouring the tumor boundary using the HE staining image. Necrotic areas were subtracted from the total area to yield the viable tumor area. Necrosis fraction was defined as the ratio of necrotic area to total area. To generate estimates of the fraction of the tumor area positive for each marker, binary images were created by imposing thresholds. The pimonidazole/Hoechst-positive area was taken to be the number of pixels that had green/blue fluorescence intensity greater than a threshold value. The PPF was then defined as the ratio of pimonidazole-positive area to viable tumor area. Similarly, the HPF was then defined as the ratio of Hoechst-positive area to viable tumor area. MVD of tumors was evaluated by counting the number of microvessels staining with CD34 per high-power field (200×) in the sections [29].

### Radiosensitizing effect of CMNa

Primary (0 Gy pre-irradiation) and recurrent esophageal tumor models (10 Gy pre-irradiation) were established and tumor volumes were monitored as above description. Mice bearing 150-200 mm^3^ tumors were randomized into 3 groups (n=10 in each group): A; control (no treatment), B; radiation alone (30 Gy in 3 weekly fractionations), C; radiation (30 Gy in 3 weekly fractionations) combined with CMNa (1 mmol/kg *i.p*. injected 60 min before irradiation). Tumor volumes were monitored and compared among groups. Relative tumor volume was defined as the ratio of measured tumor volume at each time point to the initial tumor volume before treatment.

The radiosensitizing effects was quantified by two parameters: (1) the tumor inhibition rate, which was defined as the difference between tumor volumes of radiation alone group and CMNa+radiation group divide those of radiation alone, and (2) the relative growth delay time, which was defined as the time intervals (days) needed to triple as tumor volumes at the beginning of treatments.

### Statistical analysis

Statistical analysis was performed using SPSS (Version 19.0). Data was expressed as mean values and standard deviations. Analysis of variance (ANOVA) and nonparametric tests were used to compare the tumor volume, necrosis fraction, PPF, HPF and growth delay. The comparison of multiple mean with ANOVA, after the equal check of variance, and the two-two comparisons among the means were done by LSD method. *P*<0.05 was considered statistically significant.

## Abbreviations

TBE: tumor bed effect
CMNa: sodium glycididazole
HE: hematoxylin-eosin
PPF: pimonidazole-positive fraction
HPF: Hoechst-positive fraction
MVD: microvessel density
ANOVA: analysis of variance
